# Cathartics

**Published:** 1879-10

**Authors:** 


CATHARTICS of every kind should be avoided, as they give only temporary relief. Their effect is to stimulate the action of the bowels during their operation, and, like all stimulants, they leave the parts debilitated. Injections are also to be avoided, on account of the troublesome and frequently fatal irritations they sometimes produce. The only positively safe plan to procure a regular and healthy movement of the bowels is to use "NELATON'S SUPPOSITORY." We recommend "Nelaton's" because there are no others so reliable. We know of cases where they have afforded permanent relief to persons who have suffered for years with dyspepsia produced by piles and constipation.

